# 12th International Multi Thematic Scientific Biomedical Congress (IMBMC), Nicosia, Cyprus, 2024

**DOI:** 10.1038/s41419-025-07865-w

**Published:** 2025-07-16

**Authors:** Giannis Socratous, Maria Elia, Panayiota Christodoulou, Theodora-Christina Kyriakou, Christina Kousparou, Charalambos Michaeloudes, Petros Agathaggelou, Anastasis Stephanou, Ioannis Patrikios

**Affiliations:** School od Medicine, European University Cyprus, Nicosia, Cyprus

**Keywords:** Translational research, Medical research

The 12th international multithematic biomedical congress (IMBMC) 2024, “Bio-Medical Scientific Cyprus,” took place at European University Cyprus (EUC), Nicosia, Cyprus, under the auspices of the Ministry of Health and the Cyprus Medical Association. IMBMC is an internationally recognized event, founded and established by Prof. Dr. Ioannis Patrikios, the Deputy Dean of the School of Medicine at EUC. During the 12^th^ IMBMC, Dr Thomas Sudhof (Nobel prize award in medicine, regarding the discovery on vesicle trafficking in 2013) was honored with the “Doctor Honoris Causa” award.

**Dr. Michael Papadakis**, Professor of Cardiology at St. George’s, University of customized and President of the European Association of Preventive Cardiology (EAPC), spoke on “Exercise Prescription in Athletes with Cardiomyopathy.” His research examined the challenges of advising exercise for athletes diagnosed with cardiomyopathy, therefore balancing possible risks against the cardiovascular benefits of physical activity. He discussed risk stratification criteria, genetic predisposition’s impact, and the part advanced imaging technologies play in identifying high-risk individuals. His findings emphasized the importance of individualized exercise programs, such cardiopulmonary assessments and monitoring strategies, to optimize safety and performance in athletes with cardiac problems. He continued to talk about tailored workout plans for at-risk athletes that help to maintain cardiovascular health and prevent unanticipated cardiac arrest. His work also highlighted fresh biomarkers that could assist in spotting early maladaptive responses to exercise.

Focusing on the early detection of cardiac abnormalities in athletes, Professor **Dr. Amanda Varnava**, Head of Cardiology at Imperial College NHS Trust and Consultant Cardiologist for various professional football teams, presented on “Advances in Sports Cardiology and Athlete Screening.” Her work included complex imaging and genetic testing to prevent sudden cardiac events in high-performance sports. Emphasizing the need of longitudinal monitoring in athlete health management, she discussed the challenges of differentiating pathological diseases from physiological cardiac adaptations. Her study provides interesting insights on how to optimize cardiovascular screening processes for elite athletes, so reducing the likelihood of undetected heart diseases.

**Professor Dr. Stavros G. Drakos**, keynote speaker and Professor of Medicine, Physiology, and Bioengineering at the University of Utah, presented “Myocardial Recovery: What Have We Learned and Future Directions.” His presentation focused on the mechanisms fueling myocardial recovery in heart failure patients, particularly those on mechanical circulatory support (MCS). He discussed imaging methods and novel biomarkers used to predict possible weaning from MCS and cardiac functional enhancement. His work emphasized the need of inflammation, fibrosis regression, and mitochondrial function in myocardial regeneration, therefore offering fresh therapeutic concepts for management of heart failure.

**Professor Dr. John McMurray**, keynote speaker and Professor of Medical Cardiology at Queen Elizabeth University Hospital, Glasgow, addressed “Beating Heart Failure: Recent Therapeutic Successes.” His presentation addressed the most recent device-based and pharmacological therapies, including SGLT2 inhibitors, ARNIs, and evolving gene treatments intended to improve heart failure outcomes. He discussed the results of clinical studies demonstrating how effectively these therapies reduced hospitalizations and improved survival rates. His work also highlighted ongoing studies on new heart failure biomarkers and their impact on guiding individualized treatment plans.

**Professor Dr. Randall C. Starling**, keynote speaker and Professor of Medicine at the Cleveland Clinic, addressed “Durable Mechanical Circulatory Support (MCS): Today and Tomorrow.” His work outlined advances in total artificial hearts (TAHs) and ventricular assist devices (VADs), emphasizing device durability, reduced complications, and expanded MCS implantation eligibility criteria. Stressing the need of continuous hemodynamic assessment in enhancing patient outcomes, he looked at how remote monitoring technology could be integrated into MCS management optimization.

**Professor Dr. Filippos Triposkiadis**, adjunct professor at the European University Cyprus, spoke on “Congestion in Heart Failure: From Bench to Bedside.” His research covered novel decongestion methods, including diuretic optimization and ultrafiltration, as well as the pathophysiology of venous congestion and its consequences on organ dysfunction. He examined how systemic congestion connected to declining renal and liver function in heart failure patients and recommended a more concentrated approach to volume control.

**Professor Dr. Konstantinos Drosatos** lectured on Krüppel-like factor 5: A novel myocardial element of heart failure. The rüppel-like factor 5 (KLF5), a DNA-binding transcription factor, occupies a pivotal position in cardiovascular biology as he mentioned. Initially KLF5 was identified as the intestinal-enriched KLF. Subsequent investigation has illuminated its role across various cellular processes, spanning development, metabolism, and disease. “Our research” as he explained, has revealed key insights into KLF5’s intricating relationship with cardiac muscle pathology. Those studies on diabetic cardiomyopathy (DbCM), which disrupts cardiac energetics and mitochondrial function, have identified KLF5 upregulation in diabetic hearts of humans and mice that leads to oxidative stress and cardiac lipotoxicity by stimulating NOX4 expression and promoting ceramide accumulation. Furthermore, Ischemic Heart Failure is associated with increased cardiomyocyte KLF5 in both human patients and murine models, as he discussed. Remarkably, KLF5 genetic and pharmacologic inhibition not only curtails ceramide accumulation but also ameliorates cardiac remodeling and improves heart fraction as he further explained. In summary, KLF5 represents a promising therapeutic target for cardiac disorders, impacting both heart function and metabolic pathways as he emphasized.

**Dr. Patrick Pallier**, Senior Lecturer in Neuroscience at Barts and The London School of Medicine and Dentistry, discussed “The Effects of Long-Term Leucine and Isoleucine Treatment on Blood-Brain Barrier Integrity and Behavioral and Cognitive Dysfunctions in Male BALB/c Mice.” Extended amino acid supplementation was the focus of his research in relation to blood-brain barrier integrity and its subsequent influence on cognition and behaviour. His findings emphasized the need of concentrated nutritional and pharmacological therapies to preserve brain function and provided significant fresh insights on metabolic consequences on neurodegenerative disorders.

**Professor Dr. Richard Frackowiak**, titular professor at EPFL Switzerland and keynote speaker, addressed “Clinical Neuroscience - What Does the Future Promise?” His research examined creative developments in neuroimaging, computational neuroscience, and personalized medicine. He stressed how large data analysis and artificial intelligence could change treatment and diagnosis paradigms of neurology. His work also covered new neuroprosthetic innovations and how brain-computer interfaces are enhancing rehabilitation for people with neurological disorders.

**Nobel prize winner Professor Dr. Thomas C. Südhof**, honorary keynote speaker, delivered a keynote talk on “Towards Understanding Synapse Formation.” His creative work on synaptic transmission mechanisms revealed the molecular basis of neuronal communication and plasticity. He discussed how alterations in synaptic structure lead neurodevelopmental and neurodegenerative disorders and proposed fresh intervention treatment objectives.

University of Geneva Medical School **Professor Dr. Stylianos Antonarakis** spoke on “The Determinism of Genetic Disorders: Mutation, Variability, and Environment.” His research examined how genetic mutations, epigenetic alterations, and environmental variables interacted to create genetic diseases. Offering new insights on disease prediction and prevention, he discussed the latest advances in gene-environment interactions, polygenic risk scores, and genome-wide sequencing.

**Professor Dr. Costas G. Hadjipanayis** Discussed how improved detection of brain tumors in the operating room can permit safe maximal resection of tumors. He discussed the latest advances in the intraoperative detection and resection of malignant brain tumors. He is a pioneer in the use of fluorescence-guided surgery (FGS) and robotic-assisted exoscope based surgery for brain tumors in the US. In addition, his group recently completed a multicenter clinical trial utilizing a handheld probe for detection of brain tumors by Raman spectroscopy and machine-based learning. He also discussed how he launched the first intraoperative photodynamic therapy (PDT) study in the US for treatment of brain cancer at the time of surgical removal.

“Immunotherapy of Cancer: A Dream Becoming Reality?” Presented by **Professor Dr. Agamemnon Epenetos**, Consultant in Clinical Oncology at the Harley Street Cancer Clinic, London, Imperial College London His work focused on the most recent developments in cancer immunotherapy, particularly the use of checkpoint inhibitors, CAR-T cell therapy, and tumor-targeted monoclonal antibodies. Examining the translational challenges in applying these therapies into clinical practice, he addressed tumor resistance mechanisms and combination strategies to enhance treatment efficacy.

**Professor Dr. Wolfgang F. Graier**, head of the NIKON-Center of Excellence at the Medical University of Graz, addressed “Revisiting the Dogma of Glucose-Stimulated Insulin Secretion in Pancreatic Beta Cells.” His research challenged conventional wisdom on insulin secretion mechanisms by showing various metabolic pathways influencing pancreatic beta-cell activity and their implications for diabetes treatment. He examined how mitochondrial function and intracellular calcium signaling working together affected beta-cell insulin release.

Discussions on advancements in surgical techniques and vascular interventions were led by session coordinator and Professor of Vascular Surgery at the University of Thessaly, Larissa University Hospital, **Professor Dr. Miltiadis (Miltos) Matsagkas**. His work emphasised the importance of early diagnosis and customised treatment strategies for vascular diseases by including endovascular therapies and hybrid approaches to improve patient outcomes. He provided studies on the development of new stent technologies and biodegradable scaffolds lowering long-term vascular issues. He also talked about variations in access to vascular treatments and strategies to increase patient compliance with secondary prevention policies.

**Dr. George K. Andrikopoulos**, president of the Hellenic Arrhythmia Institute, delivered an interesting talk on “New Modalities for LBB Pacing and Pulsed Field Ablation of Persistent Atrial Fibrillation.” His research examined the physiological benefits of left bundle branch pacing (LBBP) over traditional right ventricular pacing and the role of pulsed-field ablation in improving arrhythmia control with reduced myocardial damage. His presentation also addressed how artificial intelligence would direct future electrophysiological therapies.

**Professor Dr. George Malliaras** from the University of Cambridge, Department of Engineering, Cambridge, United Kingdom lectured on Bioelectronic Medicine. Neurological conditions affect one in six people, imposing significant health, economic and societal burden as he discussed. Bioelectronic medicine aims to restore or replace neurological function with the help of implantable electronic devices. The clinical application of these devices has already revolutionised the care of many patients. Deep brain stimulators for Parkinson’s disease, vagus nerve stimulators for epilepsy, spinal cord stimulators for refractory neuropathic pain and cochlear implants for hearing disorders are just some examples as he emphasized. Unfortunately, significant technological limitations prohibit these devices from reaching patients at scale: Current devices in clinical use are assembled by hand using methods that date back to the first cochlear implants developed in the late 1960s, he explained. As a result, implants are bulky, require invasive implantation procedures, elicit a pronounced foreign body response, and show poor treatment specificity and off-target effects, he continued explaining. Over the past decade, neurotechnology devices made using methods from microelectronics industry have been shown to overcome these limitations as he stated, and he proceeded discussing the state-of-the-art of these new technologies and the barriers that need to be overcome to reach patients at scale.

Last comments

Emphasizing innovation, precision medicine, and patient-centered approaches, these presentations underlined revolutionary research and clinical developments in different domains of medicine. The lectures provided insightful analysis, and the discussions provided examination of the shifting landscape of medical science, with optimism for the future of healthcare. To further promote medical breakthroughs, the speakers stressed the need of cooperation among physicians in academia, research and businesspeople in medical and pharmaceutical industry. All things considered, the talks offered a look into the hopeful future of healthcare by means of innovative technologies and individualized treatment choices.

